# Improving bleeding and thrombosis in patients with extracorporeal life support: could hematology help?

**DOI:** 10.1016/j.rpth.2026.103422

**Published:** 2026-03-24

**Authors:** Heidi J. Dalton

**Affiliations:** Department of Surgery and Clinical Pediatrics, Creighton University (Phoenix Campus), Omaha, Nebraska, USA

Despite the advances in technology, new patient populations, new medications, and multiple monitoring algorithms for anticoagulation, bleeding, and thrombosis complications remain high among those receiving extracorporeal life support (ECLS) [1,2]. Multiple consensus meetings and expert panels with rigorous evaluation of the available literature to develop “best practice” guidelines have appeared over the past several years, but none provide universally agreed upon or scientifically proven anticoagulation medications or monitoring regimens that convincingly lower bleeding and thrombosis [3,4]. Despite multiple efforts in this area, it has been of interest that the hematology physicians, who one might think would have both interest and expertise in dealing with anticoagulation, are often not included in any discussions regarding patients with ECLS. Recently, Regling et al. [5] reported the results of a survey from several organizations that deal with ECLS in an attempt to shed new light on practice and further delineate the role of hematologists in ECLS practice. While the actual number of surveys that were completed and analyzed is disappointingly low, they do add some data as to current practice. Most notably, variability in practice between centers remains high, and the use of hematology services remains low. While in some circumstances hematology may not be accessible to ECLS patients, this seems less likely than the fact that clinicians caring for patients with ECLS routinely do not recognize that hematology involvement may be useful. Additionally, to be fair, many hematology clinicians have no idea of what ECLS entails and the impact of the extracorporeal circuit on blood, platelets, and the coagulation cascade. Their practice may revolve more around primary hematology illnesses, focused on malignancies, sickle cell disease, or others. Nonetheless, education of ECLS care to our hematology colleagues may provide new insight into advances in anticoagulation, monitoring, and ways to focus research on means to reduce bleeding and thrombosis that they may bring to our multidisciplinary team.

The authors also note that there is an increased use of bivalirudin, although the reasons why a change from heparin in those centers that consider heparin a first-line drug are unclear [6]. Monitoring of bivalirudin and heparin seems to focus on activated partial thromboplastin time (aPTT)—a test that has been criticized as difficult to interpret between centers due to multiple assays, reagents, analyzers, and nomograms and that has age-related variability as well [7,8]. No test, whether aPTT, activated clotting time, or anti-Xa, has been shown to be associated with bleeding and thrombosis in children receiving heparin infusions during extracorporeal membrane oxygenation (ECMO) [9]. Use of aPTT during bivalirudin infusion becomes unreliable when aPTT levels are high, and the availability of potentially “more accurate” tests, such as dilute thrombin time, is scarce, and results are not universally accepted [6,8]. Use of more novel tests, such as viscoelastic evaluation, which seems to hold promise as they evaluate clot formation through lysis, is still practiced in less than a third of centers, and most do not use anything from the analysis other than clotting time—potentially ignoring the data on platelet and fibrinogen function that viscoelastic tests may provide.

There is also variability in the dosing of anticoagulant medications and anticoagulation laboratory testing regimens, with some centers monitoring frequently and others less. The impact of phlebotomy from laboratory testing and the need for red cell transfusion in children has been described previously, and even in adults, multiple laboratory draws can also impact the need for blood replacement—and more laboratory tests also result in increased health care costs [10,11]. It is of some interest that use of plasma-free hemoglobin, often considered a sign of hemolysis in the ECMO circuit, was reported in about 80% of patients, a percentage that is quite a bit higher than other reports. Whether the disparity is a result of the fact that the Extracorporeal Life Support Organization (ELSO) registry collects these data and many of the survey respondents were ELSO center members or the fact that this variable is actually being measured more frequently is unclear. The survey did not include any assessment of plasma-free hemoglobin results or either circuit or patient complications.

As with many surveys, the respondent rates were poor, and there are also multiple examples of how poorly survey results actually correlate with practice. As many ECMO centers do not belong to ELSO and may not be a part of those to whom the survey was aimed, the results cannot be extrapolated widely to the general ECMO community. Nonetheless, the survey does add some new information to the field, including how little hematology services are included in routine ECLS care and the continued variability in practice between centers. As variability in practice has been associated with morbidity and mortality, perhaps focusing on standardization of anticoagulation practice between sites may allow answers to questions that continue to plague our care [[11], [12], [13]].
